# Efficient Wind Speed Forecasting for Resource-Constrained Sensor Devices

**DOI:** 10.3390/s21030983

**Published:** 2021-02-02

**Authors:** Sergio Herrería-Alonso, Andrés Suárez-González, Miguel Rodríguez-Pérez, Raúl F. Rodríguez-Rubio, Cándido López-García

**Affiliations:** AtlanTTic, Universidade de Vigo, 36310 Vigo, Spain; asuarez@det.uvigo.es (A.S.-G.); miguel@det.uvigo.gal (M.R.-P.); rrubio@det.uvigo.es (R.F.R.-R.); candido@det.uvigo.es (C.L.-G.)

**Keywords:** energy harvesting, wind energy, energy management, energy prediction

## Abstract

Wind energy harvesting technology is one of the most popular power sources for wireless sensor networks. However, given its irregular nature, wind energy availability experiences significant variations and, therefore, wind-powered devices need reliable forecasting models to effectively adjust their energy consumption to the dynamics of energy harvesting. On the other hand, resource-constrained devices with limited hardware capacities (such as sensor nodes) must resort to forecasting schemes of low complexity for their predictions in order to avoid squandering their scarce power and computing capabilities. In this paper, we present a new efficient ARIMA-based forecasting model for predicting wind speed at short-term horizons. The performance results obtained using real data sets show that the proposed ARIMA model can be an excellent choice for wind-powered sensor nodes due to its potential for achieving accurate enough predictions with very low computational burden and memory overhead. In addition, it is very simple to setup, since it can dynamically adapt to varying wind conditions and locations without requiring any particular reconfiguration or previous data training phase for each different scenario.

## 1. Introduction

Wireless sensor networks (WSNs) are often composed of a large number of self-sustainable, autonomous sensor nodes that operate under stringent resource restrictions, such as limited battery, communication, storage, and computing capabilities. Frequently, sensor nodes use some of the currently available energy harvesting (EH) technologies to obtain an uninterrupted power supply for all practical purposes [1]. However, EH nodes must commonly apply an energy management policy to consume the harvested energy effectively and ensure long-term operation due to the high intermittency and irregular nature of ambient energy sources, such as sunlight or wind [2].

**Figure 1:**
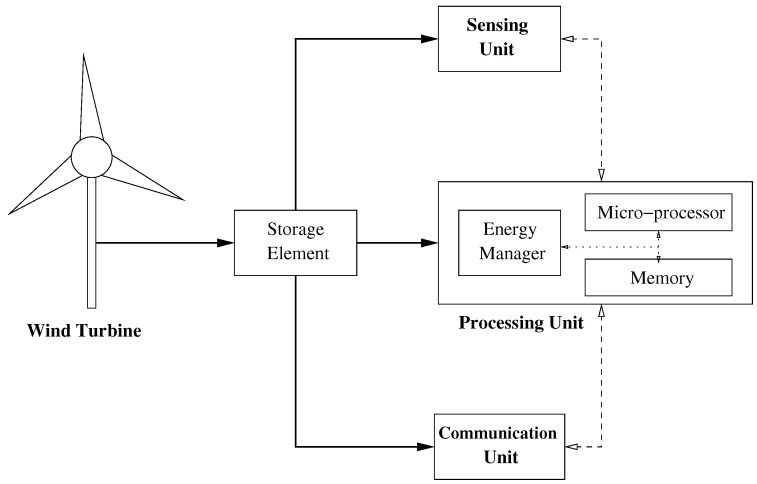
Block diagram of a wind-powered energy harvesting (EH) node. Solid (dotted) lines represent energy (data) transfer.

Wind energy is one of the most popular and valuable ambient energy sources due to its high power intensity and economic competitiveness. Figure 1 shows the block diagram of a wind-powered EH node. It includes a wind turbine that converts wind energy into electrical energy. Most wind turbines usually work in the following manner. The wind turns the turbine blades, which spin a shaft, thus converting wind energy into low speed rotational energy. To speed up rotation, the low-speed shaft is connected to a high-speed one through a gearbox. Because the high-speed shaft is attached to a coil of copper surrounded by a magnetic field, a current is then induced in the copper coil.

Electrical energy that is generated by the wind turbine can be stored, for example, in a rechargeable battery or a capacitor. The storage element powers the processing, sensing and communication units. The sensing unit performs the sensor functionality (not necessarily wind-related), while the communication unit transmits and receives measures and control data. The processing unit stores and handles sensed and received data. It also implements the energy management scheme required to schedule processing, sensing, and communication functions optimally from an energy point of view, as shown in the block diagram [3,4].

Wind speed has a great impact on the amount of energy that is generated by wind turbines. In fact, the amount of energy that can be theoretically obtained from the wind is proportional to the cube of wind speed [5], although, in practice, the power output of a specific wind turbine and the wind speed are usually related following a sigmoid power curve that is given by the manufacturer [6]. Certainly, wind speed is highly dynamic, so the energy that is available to a wind-powered device fluctuates significantly, even within short periods of time. Therefore, the energy management scheme implemented in a wind-powered node requires an efficient forecasting model that accurately predicts wind speed in the near future (from a few minutes to a few hours) to effectively adapt energy consumption to the dynamics of EH and avoid forthcoming energy shortages [7].

Many different wind speed/power forecasting models have been proposed in recent years [8,9]. According to their forecasting approach, existing models can be classified into three different categories: physical, statistical, and hybrid models. Physical methods model wind power while taking into account some physical specifications of wind turbines, local terrain, and farm layouts, as well as meteorological data that were obtained from numerical weather prediction (NWP) [10,11,12]. Because of the high latency and computational cost of NWP, physical methods have limited utility for short-term predictions, although they can perform well for long forecasting horizons (greater than 6 h). Different from them, statistical methods model wind speed/power as a stochastic process formed from the available time series of historical data [13,14,15,16,17,18,19,20,21,22,23,24,25,26,27,28,29,30,31,32,33,34,35]. These models have lower complexity and latency than the physical ones, so they are preferred for short-term forecasting horizons. Finally, hybrid models try to benefit from the best features of both physical and statistical models combining meteorological conditions forecasts (such as temperature, humidity, or atmospheric pressure) with available time series [36,37,38]. However, because hybrid models also require NWP, they are also inadequate for short-term forecasting.

Wind-powered sensor nodes must work in WSNs with limited hardware capacities and under severe resource restrictions. Therefore, they require simple and effective forecasting schemes for their short-term wind predictions. In this paper, we present a new efficient ARIMA-based forecasting model to predict wind speed at short-term horizons (from 10 min to 1 h). The performance results that were obtained using real data sets confirm that the proposed ARIMA model can be an excellent choice for resource-constrained sensor devices, due to its capacity to achieve accurate enough predictions using a very small amount of memory and just performing a few straightforward operations. In addition, and different from the previous ARIMA models, the management and setup of the proposed method is very simple, since it is able to dynamically adapt to changing wind conditions and locations without requiring any reconfiguration or previous data training phase for each different scenario.

The rest of the paper is organized, as follows. Related work is reviewed in Section 2. Section 3 briefly describes the different forecasting models that have been evaluated in the paper. In Section 4, we present a novel ARIMA-based model that is specifically designed to forecast future values of a wind speed time series and in Section 5 we describe how it can be implemented in a light way. In Section 6, we then compare the performance of the different forecasting methods in several scenarios while using real data sets. Finally, the main conclusions are laid out in Section 7.

## 2. Related Work

As previously explained, statistical methods should be preferred for short-term predictions due to their lower latency and computational cost. These methods use available time series of historical wind speed and/or power data to make predictions. For example, some of them approach the forecasting problem while using conventional statistical methods, including Autoregressive Integrated Moving Average (ARIMA) models [13,14,15,16,17,18], Bayesian regression [19,20,21], or Kalman filtering [22,23,24].

Differently, most recent statistical methods employ some modern artificial intelligence and machine learning tools, due to their effectiveness in forecasting non-linear time series. In particular, quantile regression [25,26], neural networks [27,28,29,30], and support vector machines [31,32,33,34,35] are widely used in this context. It is worth remarking that most of these prediction schemes decompose the original non-stationary time series into several relatively stationary components to then apply the most adequate statistical model to each of them. Eventually, the final prediction is obtained by adding up all of the individual forecasting results [39].

However, there exist a few drawbacks that should be considered when using machine learning techniques in WSNs [40]. Firstly, they have to perform a great number of complex operations to forecast wind data. Specifically, the higher the required accuracy, the higher the computational burden and, hence, energy consumption. In addition, they require a large data set of samples to be trained and fit their configuration parameters for a particular location. Consequently, although these schemes may be a good choice for providing accurate medium and long-term predictions at wind farms, they are not adequate for resource-constrained, possibly portable, sensor devices.

A different class of statistical models make predictions carefully combining the latest measured values with those that were observed at the same times on previous days (EWMA [7], WCMA [41], QL-SEP [42], LINE-P [43], and D-WCMA [44]) or on the most similar past days to the current one (UD-WCMA [44] and Pro-Energy [45]). These models are simpler than those that are based on machine learning tools and they are especially suited when daily weather patterns are observed.

In this paper, we present a new ARIMA-based forecasting scheme that is especially suitable for wind-powered sensor devices with limited resources. Contrary to previous ARIMA models, the proposed method is flexible enough to dynamically adapt to varying wind conditions and/or locations without requiring any reconfiguration or previous data training phase for each different scenario. Furthermore, it can be implemented in a very light way, thus providing accurate enough short-term predictions with minimal computational load and memory overhead. In order to test its effectiveness, we compared it with the straightforward persistence model, which is usually used as a benchmark for wind speed forecasting, and with the D-WCMA and Pro-Energy schemes, due to their relative simplicity and good performance.

## 3. Forecasting Models

In this section, we present the forecasting models that have been evaluated in the paper. We assume that wind speed observations are available at discrete, equally spaced intervals of time, forming a wind speed time series $\left\{s_{0},s_{1},\ldots ,s_{n}\right\}$.

### 3.1. Persistence Model

The persistence model straightforwardly estimates the wind speed for future timeslot n+h, with h∈{1,2,…}, as the wind speed that was observed during the last timeslot *n*:

(1) $${\hat{s}}_{n+h}=s_{n}.$$

This simple method provides good predictions at short-term horizons and it is commonly used as a benchmark model for wind speed forecasting due to the high correlation between wind speed samples that are close in time. However, the accuracy of this model decreases considerably with the distance to the forecasting horizon.

### 3.2. ARIMA Model

The ARIMA (AutoRegressive Integrated Moving Average) model is a generalization of the ARMA (AutoRegressive Moving Average) model, which is recommended when the time series show evidence of non-stationarity, as is the case for the wind speed data. An ARIMA (p,d,q) model for the wind speed time series data is given by

(2) $${▽}^{d}s_{n}-\varphi_{1}{▽}^{d}s_{n-1}-\cdots -\varphi_{p}{▽}^{d}s_{n-p}=\varepsilon_{n}-\theta_{1}\varepsilon_{n-1}-\cdots -\theta_{q}\varepsilon_{q-1},$$

where *p* is the order of the autoregressive (AR) model, *q* is the order of the moving-average (MA) model, and *d* is the degree of differencing, which is the number of times that consecutive raw values have been subtracted to eliminate the non-stationarity:

(3) $$\begin{array}{cc} {▽}^{1}s_{n} & =▽s_{n}=s_{n}-s_{n-1},\\ {▽}^{d}s_{n} & ={▽}^{d-1}s_{n}-{▽}^{d-1}s_{n-1},\;\forall d\geq 2. \end{array}$$

In the ARIMA model, the parameters $\varphi_{1},\varphi_{2},\ldots ,\varphi_{p}$ of the corresponding AR model set how much the previous terms contribute to the current value, while the parameters $\theta_{1},\theta_{2},\ldots ,\theta_{q}$ of the corresponding MA model set how much the error terms, $\varepsilon_{n}$, contribute to the current value. The error terms are assumed to form a white noise process with zero mean and constant variance. Note that the conventional AR (*p*), MA (*q*), and ARMA (p,q) models are equivalent to ARIMA (p,0,0), ARIMA (0,0,q), and ARIMA (p,0,q) models, respectively, and that the persistent model can be characterized as a simple ARIMA (1,0,0) model with $\varphi_{1}=1$.

### 3.3. Pro-Energy Model

The Pro-Energy scheme maintains a time series that encompasses those samples that were obtained during the past *D* days. Assuming that *N* observations are available for each day, the time series is organized as a pool $P=\{p_{1},p_{2},\ldots ,p_{D}\}$ of *D* profiles, each one containing the *N* values that were measured during each of the timeslots of a given past day. Note that, therefore, the last sample $s_{n}$ corresponds to the *l*-th sample, with l=nmodN, of the current day.

The Pro-Energy model computes the expected value for future timeslot n+h, as

(4) $${\hat{s}}_{n+h}=\gamma_{h}\;s_{n}+\left(1-\gamma_{h}\right)\;{\bar{s}}_{l+h}^{P}\;,$$

where ${\bar{s}}_{l+h}^{P}$ is the average value of the samples that correspond to the (l+h)-th timeslot of the *P* most similar profiles of the pool P and $\gamma_{h}$ is the correlation factor that determines the significance of the last sample $s_{n}$ when making predictions:

(5) $$\gamma_{h}={\left\{\alpha \left(1-\frac{h-1}{G}\right)\right\}}^{+},$$

where α is a weighting factor, 0≤α≤1, and *G* is a parameter representing the number of timeslots in the future that presumably show a strong correlation with the value that was observed during the last timeslot. Clearly, the weight that is associated to the last sample progressively decreases as predictions go away in time.

In order to compute ${\bar{s}}_{l+h}^{P}$, the similarity with the current day for each profile $p_{i}\in P$ must first be estimated. This similarity is computed for each profile $p_{i}$, i=1,…,D, as the mean absolute error (MAE) over the previous *K* timeslots of each day:

(6) $${\mathrm{MAE}}_{K}\left(p_{i}\right)=\sum_{k=0}^{K-1}\frac{1}{K}\left|s_{n-k}-s_{l-k}^{p_{i}}\right|\;,$$

where $s_{l-k}^{p_{i}}$ is the (l−k)-th sample in profile $p_{i}$. Subsequently, if we assume that $\left\{p_{1},p_{2},\ldots ,p_{D}\right\}$ is the ordered set of profiles based on their similarity with the current day, the weighted average value ${\bar{s}}_{l+h}^{P}$ is computed as

(7) $${\bar{s}}_{l+h}^{P}=\left\{\begin{array}{cc} s_{l+h}^{p_{1}}\;, & \;\mathrm{if}\;P=1,\\ \frac{1}{P-1}\sum_{i=1}^{P}w_{i}\;s_{l+h}^{p_{i}}\;, & \;\mathrm{if}\;P>1, \end{array}\right.$$

where P<D is the number of profiles combined and

(8) $$w_{i}=1-\frac{{\mathrm{MAE}}_{K}\left(p_{i}\right)}{\sum_{j=1}^{P}{\mathrm{MAE}}_{K}\left(p_{j}\right)},\;i=1,\ldots ,P\;.$$

### 3.4. D-WCMA Model

As Pro-Energy, the D-WCMA (Dynamic Weather Condition Moving Average) scheme also maintains the samples obtained during the past *D* days, organized as a pool $P=\{p_{1},p_{2},\ldots ,p_{D}\}$ of *D* profiles. Again, each profile contains the *N* values that were measured during each of the timeslots of the corresponding past day and the last sample $s_{n}$ corresponds to the *l*-th sample, with l=nmodN, of the current day. With D-WCMA, the value that is predicted for future timeslot n+h is computed as

(9) $${\hat{s}}_{n+h}=\alpha_{l+h}\;s_{n}+\left(1-\alpha_{l+h}\right)\;{\mathrm{GAP}}_{K}\;{\bar{s}}_{l+h}\;,$$

where $\alpha_{l+h}$ is the adaptive weight of the last sample in the prediction for timeslot n+h, GAP${}_{K}$ is a factor that scales the disparity of the last samples with respect to those that were obtained in the previous days over a time window of *K* timeslots and ${\bar{s}}_{l+h}$ is the average value of the (l+h)-th samples in the previous days:

(10) $${\bar{s}}_{l+h}=\frac{1}{D}\sum_{i=1}^{D}s_{l+h}^{p_{i}}\;,$$

where $s_{l+h}^{p_{i}}$ is the (l+h)-th sample in profile $p_{i}$.

The weighting factor $\alpha_{l+h}$ estimates the predictability level of the future value from the variations in the samples of the previous days. It is dynamically configured as

(11) $$\alpha_{l+h}=\frac{1}{2}\frac{\sigma_{l+h}}{\sigma_{l+h}+\sigma_{l+h}^{\prime }}\;,$$

where

(12) $$\begin{array}{cc} \sigma_{l+h} & =\sqrt{\frac{1}{D}\sum_{i=1}^{D}{\left(s_{l+h}^{p_{i}}-{\bar{s}}_{l+h}\right)}^{2}}\;,\\ \sigma_{l+h}^{\prime } & =\sqrt{\frac{1}{D}\sum_{i=1}^{D}{\left(\Delta s_{l+h}^{p_{i}}-{\bar{\Delta s}}_{l+h}\right)}^{2}}\;,\\ \Delta s_{l+h}^{p_{i}} & =s_{l+h}^{p_{i}}-s_{l}^{p_{i}}\;,\\ {\bar{\Delta s}}_{l+h} & =\frac{1}{D}\sum_{i=1}^{D}\Delta s_{l+h}^{p_{i}}\;. \end{array}$$

Note that $\sigma_{l+h}$ is the standard deviation of the (l+h)-samples in the preceding days, whereas $\sigma_{l+h}^{\prime }$ is the standard deviation of the variations between the *l* and (l+h)-samples on those days. Finally, the GAP factor is computed as a normalized weighted average of the ratio between the last samples and the average value of the samples in the previous days along the last *K* timeslots:

(13) $${\mathrm{GAP}}_{K}=\frac{\sum_{k=1}^{K}\frac{k}{K}\frac{s_{n-K+k}}{{\bar{s}}_{l-K+k}}}{\sum_{k=1}^{K}\frac{k}{K}}=\frac{2}{K(K+1)}\sum_{k=1}^{K}k\;\frac{s_{n-K+k}}{{\bar{s}}_{l-K+k}}\;.$$

## 4. Forecasting Wind Speed Using an ARIMA Model

We now show how an ARIMA model may be used to forecast future values of wind speed time series while using the well-known Box–Jenkins method [46].

### 4.1. Data Sets Description

We selected three representative real data sets to identify those ARIMA models that fit better with wind speed time series, each one covering a one year long period, obtained from the National Renewable Energy Laboratory (NREL) at three different locations: the Oak Ridge National Laboratory (ORNL) at Oak Ridge, Tennessee [47], the National Wind Technology Center (NWTC) at Boulder, Colorado [48], and the Solar Radiation Research Laboratory (SRRL) at Golden, Colorado [49]. Original data sets contain one sample per minute of the wind speed at the given location, so we built the wind speed time series computing the average wind speed at each 10 min interval (the length of prediction intervals).

### 4.2. Model Identification

**Figure 2:**
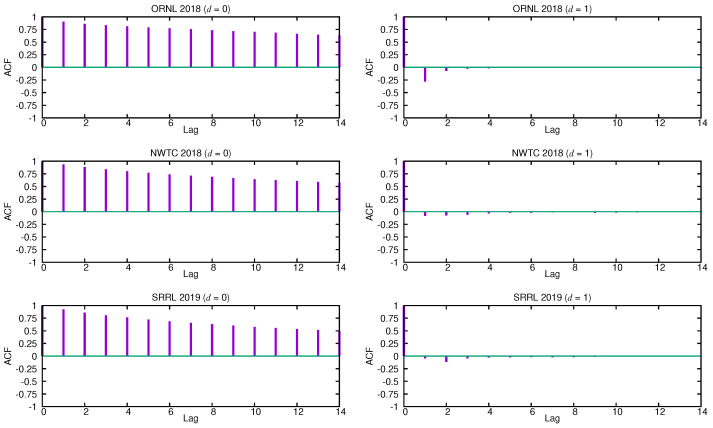
Autocorrelation functions (ACFs) for d=0 and d=1.

The first task is to identify an appropriate subclass of models from the general ARIMA family that may be used to represent a wind speed time series, that is, find out suitable values of *p*, *d* and *q* for this particular class of time series. The autocorrelation function (ACF) and the partial autocorrelation function (PACF) are the main tools for guessing the form of the model. The ACF quantifies the similarity between the terms of a time series as a function of the lag between them, while the PACF gives the partial correlation of a time series with its own lagged values after removing the effect of any correlations due to the terms at shorter lags. Figure 2 shows the estimated ACF functions for the three selected time series without differencing (d=0) and with a differencing step d=1. In [46], it is proven that the degree of differencing that is necessary to achieve stationarity is reached when the ACF function dies out quickly. The estimated ACF functions without differencing (d=0) of all the given time series fall off slowly and almost linearly, so the underlying stochastic process must be treated as non-stationary, as shown in the figure. However, the autocorrelation coefficients for d=1 become rapidly negligible after the first lags. This suggests that these time series might be well described by an ARIMA (p,1,q) process.

**Figure 3:**
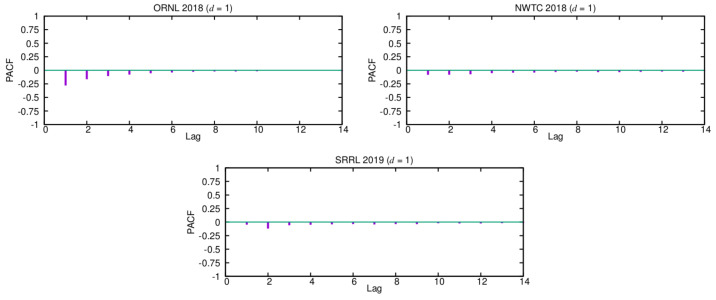
Partial autocorrelation functions (PACFs) for d=1.

Once deciding what degree of differencing should be chosen, we next use the estimated ACF and PACF functions of the corresponding differenced series to select the appropriate orders, *p* and *q*, for them. In order to find the order *q* of the MA term, we can look at the ACF plots for d=1. Because only a few of the first lags have significant autocorrelation values, setting the order q=1 or q=2 is reasonable. Additionally, note that most of the values are close to zero, thus remarking the essentially random nature of these time series. Similarly, to set the order *p* of the AR term, we can use the PACF functions that were estimated for d=1, as shown in Figure 3. Again, only the first PACF coefficients have significant values, so the order *p* should be fixed to a small value (p≤2).

Finally, to determine the most adequate specific ARIMA model for wind speed time series, we take the relationship between the two first autocorrelation coefficients into consideration. Clearly, there are only two possible scenarios. If the first autocorrelation coefficient is much more significant than the second one, as is the case for the ORNL series, wind speed series might be described by an ARIMA (1,1,1) process given by the following difference equation:

(14) $$▽s_{n}-\varphi_{1}▽s_{n-1}=\varepsilon_{n}-\theta_{1}\varepsilon_{n-1}.$$

On the contrary, if the second autocorrelation coefficient is more significant than (or comparable to) the first one, as it happens with both the NWTC and SRRL series, then the wind speed series might be better described by an ARIMA (0,1,2) process that is given by

(15) $$▽s_{n}=\varepsilon_{n}-\theta_{1}\varepsilon_{n-1}-\theta_{2}\varepsilon_{n-2}.$$

Recall that, in our context, it is important to employ the smallest possible number of parameters required for adequate representation. Consequently, we consider that an ARIMA (1,1,1) or an ARIMA (0,1,2) process might alternatively describe wind speed time series, depending on the relationship between the two first autocorrelation coefficients. We have analyzed a lot of wind speed time series from many different locations and found that they all exhibit similar correlation characteristics to any of the three series described in the paper.

### 4.3. Parameters Estimation

Once it is assumed that wind speed time series will be described by an ARIMA (p,1,q) process, we then need to estimate the corresponding AR ($\varphi_{1},\ldots ,\varphi_{p}$) and MA ($\theta_{1},\ldots ,\theta_{q}$) parameters. For example, $\varphi_{1}$ and $\theta_{1}$ must be computed if we were considering an ARIMA (1,1,1) process. As shown in [46], these parameters can be calculated by solving the following system of two equations and two variables:

(16) $$\begin{array}{cc} \rho_{2} & =\rho_{1}\varphi_{1},\\ \rho_{1} & =\frac{\left(1-\theta_{1}\varphi_{1}\right)\left(\varphi_{1}-\theta_{1}\right)}{1+\theta_{1}^{2}-2\varphi_{1}\theta_{1}}, \end{array}$$

where $\rho_{1}$ and $\rho_{2}$ are the first and second autocorrelation coefficients, respectively. Both of the parameters, $\varphi_{1}$ and $\theta_{1}$, must take a value within the range (−1,1). On the other hand, if an ARIMA (0,1,2) process is considered, then we must compute $\theta_{1}$ and $\theta_{2}$ parameters. In this case, the required parameters are the solutions to the following system of two equations:

(17) $$\begin{array}{cc} \rho_{1} & =\frac{-\theta_{1}+\theta_{1}\theta_{2}}{1+\theta_{1}^{2}+\theta_{2}^{2}},\\ \rho_{2} & =\frac{-\theta_{2}}{1+\theta_{1}^{2}+\theta_{2}^{2}}, \end{array}$$

and they must fulfill the following conditions: $-1<\theta_{2}<1$, $\theta_{1}+\theta_{2}<1$, and $\theta_{2}-\theta_{1}<1$.

### 4.4. Making Predictions

Once the model is fitted to actual data, we can then forecast a future ${\hat{s}}_{n+h}$ value at timeslot *n* in terms of the difference Equations (14) and (15). From them, it follows that

(18) $$\left\{\begin{array}{cc} {\hat{▽s}}_{n+h}=\varphi_{1}▽s_{n+h-1}-\theta_{1}\varepsilon_{n+h-1}+\varepsilon_{n+h},\; & \mathrm{with}\;\mathrm{an}\;\mathrm{ARIMA}\;(1,1,1)\;\mathrm{model},\\ {\hat{▽s}}_{n+h}=-\theta_{1}\varepsilon_{n+h-1}-\theta_{2}\varepsilon_{n+h-2}+\varepsilon_{n+h},\; & \mathrm{with}\;\mathrm{an}\;\mathrm{ARIMA}\;(0,1,2)\;\mathrm{model}, \end{array}\right.$$

and, since $▽s_{n+h}=s_{n+h}-s_{n+h-1}$, we get

(19) $$\left\{\begin{array}{cc} {\hat{s}}_{n+h}=\left(1+\varphi_{1}\right)s_{n+h-1}-\varphi_{1}s_{n+h-2}-\theta_{1}\varepsilon_{n+h-1}, & \mathrm{with}\;\mathrm{an}\;\mathrm{ARIMA}\;(1,1,1)\;\mathrm{model},\\ {\hat{s}}_{n+h}=s_{n+h-1}-\theta_{1}\varepsilon_{n+h-1}-\theta_{2}\varepsilon_{n+h-2}, & \mathrm{with}\;\mathrm{an}\;\mathrm{ARIMA}\;(0,1,2)\;\mathrm{model}, \end{array}\right.$$

Note that, for prediction horizons h>1, these models require samples $s_{n+j}$, with j≥1, which have not yet been observed, so they are replaced by their respective forecasts ${\hat{s}}_{n+j}$. Consequently, $\varepsilon_{n+j}=▽s_{n+j}-{\hat{▽s}}_{n+j}$, for j≥1, are replaced by zeroes.

## 5. Adaptive ARIMA Implementation

In order to forecast future wind speed observations using the proposed ARIMA models, we must solve the system of Equation (16) or (17) to obtain estimates of the corresponding AR and MA parameters, as explained in Section 4.3. Recall that both of the systems depend on $\rho_{1}$ and $\rho_{2}$, the first and second autocorrelation coefficients, so these coefficients must be previously estimated. Given our finite time series $▽s_{1},▽s_{2},\ldots ,▽s_{n}$ of *n* observations, it is well known that the most straightforward estimate of the *k*-th autocorrelation coefficient $\rho_{k}$ is

(20) $${\hat{\rho }}_{k}=\frac{{\hat{\gamma }}_{k}}{{\hat{\gamma }}_{0}},$$

where

(21) $${\hat{\gamma }}_{k}=\frac{1}{n}\sum_{i=1}^{n-k}\left(▽s_{i}-\bar{▽s}\right)\left(▽s_{i+k}-\bar{▽s}\right),\;k=0,1,2,\ldots ,$$

is the estimate of the autocovariance at lag *k* and $\bar{▽s}$ is the mean of the time series [50]. Fortunately, because $\bar{▽s}=0$, the computation of the autocovariance coefficients ${\hat{\gamma }}_{k}$ of the differenced time series can be greatly simplified:

(22) $${\hat{\gamma }}_{k}=\frac{1}{n}\sum_{i=1}^{n-k}▽s_{i}▽s_{i+k},\;k=0,1,2,\ldots ,$$

and, therefore, the autocorrelation coefficient $\rho_{k}$ can be easily estimated as

(23) $${\hat{\rho }}_{k}=\frac{\sum_{i=1}^{n-k}▽s_{i}\;\cdot \;▽s_{i+k}}{\sum_{i=1}^{n}▽s_{i}^{2}},\;k=0,1,2,\ldots$$

### 5.1. Parameters Estimation

In Section 4.2 we found that wind speed time series can be alternatively described by an ARIMA (1,1,1) or an ARIMA (0,1,2) process. The selection of the most adequate model for a given series is driven by the relative weight of its two first autocorrelation coefficients. Thus, if $|{\hat{\rho }}_{1}\left|>\right|{\hat{\rho }}_{2}|$, i.e., if $|{\hat{\gamma }}_{1}\left|>\right|{\hat{\gamma }}_{2}|$, then the ARIMA (1,1,1) model must be selected and the corresponding $\varphi_{1}$ and $\theta_{1}$ parameters must be computed solving the equation system (16). From the first equation in this system and (20), we get that the $\varphi_{1}$ parameter can be easily estimated as

(24) $${\hat{\varphi }}_{1}=\frac{{\hat{\rho }}_{2}}{{\hat{\rho }}_{1}}=\frac{{\hat{\gamma }}_{2}/{\hat{\gamma }}_{0}}{{\hat{\gamma }}_{1}/{\hat{\gamma }}_{0}}=\frac{{\hat{\gamma }}_{2}}{{\hat{\gamma }}_{1}},$$

and, then, ${\hat{\theta }}_{1}$ can be determined just solving the second equation in system (16). However, if $\rho_{1}$ (and, therefore, $\rho_{2}$) takes a small value, as is the case with wind speed time series (see Figure 2), the solution to this equation can be well approximated as ${\hat{\theta }}_{1}\approx {\hat{\varphi }}_{1}-{\hat{\rho }}_{1}$.

Conversely, when $|{\hat{\rho }}_{1}\left|<\right|{\hat{\rho }}_{2}|$, i.e., $|{\hat{\gamma }}_{1}\left|<\right|{\hat{\gamma }}_{2}|$, the ARIMA (0,1,2) model is more adequate, so, in this case, the equation system (17) must be solved in order to estimate the corresponding $\theta_{1}$ and $\theta_{2}$ parameters. Fortunately, solving this system again becomes straightforward when both $\rho_{1}$ and $\rho_{2}$ coefficients take small values. Under these conditions, it can be proved that the valid solutions to system (17) can be well approximated as ${\hat{\theta }}_{1}\approx -{\hat{\rho }}_{1}$ and ${\hat{\theta }}_{2}\approx -{\hat{\rho }}_{2}$. Therefore, the predictor must just perform the following simple operations to estimate the ARIMA parameters:

- if $|{\hat{\gamma }}_{1}\left|>\right|{\hat{\gamma }}_{2}|$ then ARIMA (1,1,1)
  1. ${\hat{\varphi }}_{1}={\hat{\gamma }}_{2}/{\hat{\gamma }}_{1}$
  $-{\hat{\theta }}_{1}={\hat{\gamma }}_{1}/{\hat{\gamma }}_{0}-{\hat{\varphi }}_{1}$
- else ARIMA (0,1,2)
  $-{\hat{\theta }}_{1}={\hat{\gamma }}_{1}/{\hat{\gamma }}_{0}$
  $-{\hat{\theta }}_{2}={\hat{\gamma }}_{2}/{\hat{\gamma }}_{0}$

In practice, the ARIMA parameters do not have to be updated with every new value. In fact, if new observations are obtained at intervals of 10 min, we propose updating the ARIMA parameters every 36 new samples, i.e., just every six hours (shorter updating intervals have shown negligible improvements in performance). Finally, at the initial system setup, the predictor can be initialized to perform as the persistence scheme during the first operating period, i.e., as an ARIMA (1,1,1) model with ${\hat{\varphi }}_{1}={\hat{\theta }}_{1}=0$, or as an ARIMA (0,1,2) model with ${\hat{\theta }}_{1}={\hat{\theta }}_{2}=0$. Certainly, this implies that early forecasts will presumably be less accurate, so the energy management scheme should be more cautious during the first operating periods.

### 5.2. Making Predictions

From (18), and assuming that the ARIMA (0,1,2) model is applied ($|{\hat{\gamma }}_{1}\left|<\right|{\hat{\gamma }}_{2}|$), it easily follows that the next value of the wind speed time series, ${\hat{s}}_{n+1}$, can be forecasted at timeslot *n* just performing the following simple operations:

1. $s_{n}\leftarrow$ last observed wind speed value
2. $▽s_{n}=s_{n}-s_{n-1}$
3. ${\hat{▽s}}_{n+1}=-{\hat{\theta }}_{1}\;\cdot \;\left(▽s_{n}-{\hat{▽s}}_{n}\right)-{\hat{\theta }}_{2}\;\cdot \;\left(▽s_{n-1}-{\hat{▽s}}_{n-1}\right)$
4. ${\hat{s}}_{n+1}=s_{n}+{\hat{▽s}}_{n+1}$
5. ${\hat{▽s}}_{n-1}\leftarrow {\hat{▽s}}_{n}$
6. ${\hat{▽s}}_{n}\leftarrow {\hat{▽s}}_{n+1}$
7. ${\hat{\gamma }}_{0}$ +$=▽s_{n}\;\cdot \;▽s_{n}$
8. ${\hat{\gamma }}_{1}$ +$=▽s_{n}\;\cdot \;▽s_{n-1}$
9. ${\hat{\gamma }}_{2}$ +$=▽s_{n}\;\cdot \;▽s_{n-2}$
10. $▽s_{n-2}\leftarrow ▽s_{n-1}$
11. $▽s_{n-1}\leftarrow ▽s_{n}$
12. $s_{n-1}\leftarrow s_{n}$

Note that the prediction is obtained in step 4. In the next steps, we just update the state variables that are required for making predictions and estimating ARIMA parameters.

In the case that the ARIMA (1,1,1) model must be applied, the same operations will be performed just replacing the third one by the following one:

3. ${\hat{▽s}}_{n+1}={\hat{\varphi }}_{1}\;\cdot \;▽s_{n}-{\hat{\theta }}_{1}\;\cdot \;\left(▽s_{n}-{\hat{▽s}}_{n}\right)$

Additionally, note that, to compute predictions for more distant horizons, these operations must be repeated several times just replacing the required, but not yet observed, samples by their respective forecasts.

Finally, it should be noted that, in the case that the sensor provides invalid wind speed measures, this model could continue making predictions just replacing the corrupted samples by their respective forecasts. If sensor errors occur occasionally, their effects will be negligible, since, as we will show in the following section, this model provides accurate enough predictions in the short term.

## 6. Evaluation

The persistence, Pro-Energy, D-WCMA, and ARIMA models have been applied to the three wind speed traces that are described in Section 4.1 using an open-source in-house simulator [51]. We evaluated the accuracy of these forecasting models for prediction horizons from h=1 to 6 (from 10 min–60 min) in terms of the mean absolute error (MAE) of their predictions:

(25) $$\mathrm{MAE}=\frac{\sum |s_{n+h}-{\hat{s}}_{n+h}|}{\;\mathrm{number}\;\mathrm{of}\;\mathrm{predictions}\;}\;.$$

Note that, contrary to the proposed ARIMA model, the computational overhead that is introduced by Pro-Energy and D-WCMA models (and, consequently, the precision of their forecasts) depends on how their main parameters are configured. We configured both of the models with three different overhead levels, as shown in Table 1. For Pro-Energy, recall that *D* is the number of previous days stored in the pool, *K* is the number of previous samples used to estimate similarity between profiles, and *P* is the amount of profiles that are combined to obtain the average value at the future timeslot. When computing the correlation factor γ, *G* was set to 15 (2.5 h), while the weighting factor α was configured with the optimal value for each simulated scenario, thus avoiding any eventual bias towards the proposed model. Regarding D-WCMA, *D*, again, is the amount of stored profiles and *K* is the number of past samples considered when computing the GAP factor.

### 6.1. Performance Comparison

**Figure 4:**
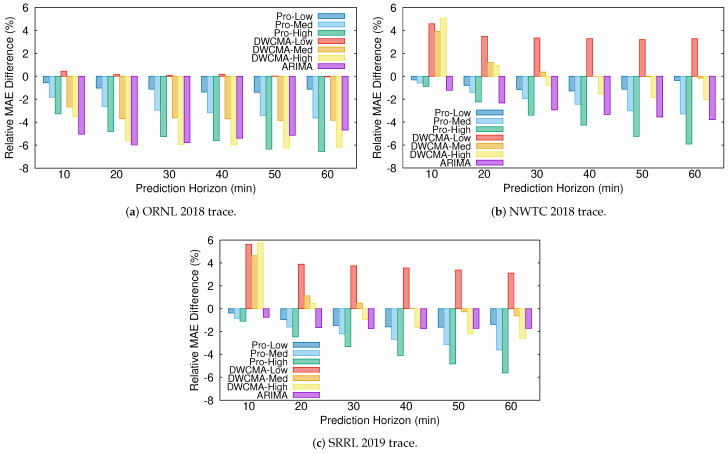
Relative mean absolute error (MAE) difference (persistence model used as baseline).

Figure 4 shows the relative difference between the MAE that was obtained with each of the models and that obtained with the persistence one for each of the wind speed traces:

(26) $$\mathrm{Relative}\;\mathrm{MAE}\;\mathrm{Difference}=\frac{{\mathrm{MAE}}_{\mathrm{model}}-{\mathrm{MAE}}_{\mathrm{persistence}}}{{\mathrm{MAE}}_{\mathrm{persistence}}}.$$

As expected, Pro-Energy and D-WCMA obtain more accurate predictions, as they are configured with higher overhead settings. In addition, their accuracy with respect to the persistence model increases with the distance to the prediction horizon. On the other hand, for the shortest prediction horizons (10–20 min), the proposed ARIMA model is able to achieve the most precise estimations in both the ORNL and NWTC traces, and only in the SRRL trace Pro-Energy with the highest overhead is able to provide slightly more accurate predictions. For the most distant horizons, the ARIMA model still provides satisfactory predictions and only Pro-Energy and D-WCMA achieve better predictions consistently when configured with the highest overhead settings. Therefore, we can affirm that the ARIMA-based model is a good alternative for wind speed forecasting in resource-constrained EH devices, since it is able to obtain predictions with an accuracy that is comparable to those obtained by Pro-Energy or D-WCMA at their highest overhead settings, but with a much lower computational burden. Finally, note that all of the forecasting schemes obtain their best performance with the ORNL trace, since, as it can be inferred from the ACF and PACF functions (see Figure 2 and Figure 3), this is the series with less randomness.

We also applied the UD-WCMA model [44], an enhanced yet more complex variant of D-WCMA, to the three selected wind speed traces. We found that, despite its considerably higher overhead, it provides very similar results to those that were obtained with D-WCMA at the shortest prediction horizons (10–40 min). Only for prediction horizons of 50–60 min, UD-WCMA is able to obtain slightly better predictions than D-WCMA, with improvements of around 0.5% in the relative MAE difference. Therefore, we decided not to include these results in the article, so as not to excessively clutter the graphs.

### 6.2. Optimistic Forecasting

As previously stated in the introduction, the main mission of the forecasting scheme in resource-constrained EH nodes is to help the energy management policy to effectively adapt energy consumption to the dynamics of EH, thus avoiding forthcoming energy shortages. However, the risk of suffering an energy shortage can be seriously increased if the forecasting scheme overestimates future energy availability, since EH nodes may then be allowed to spend more energy than they will really have at their disposal.

**Figure 5:**
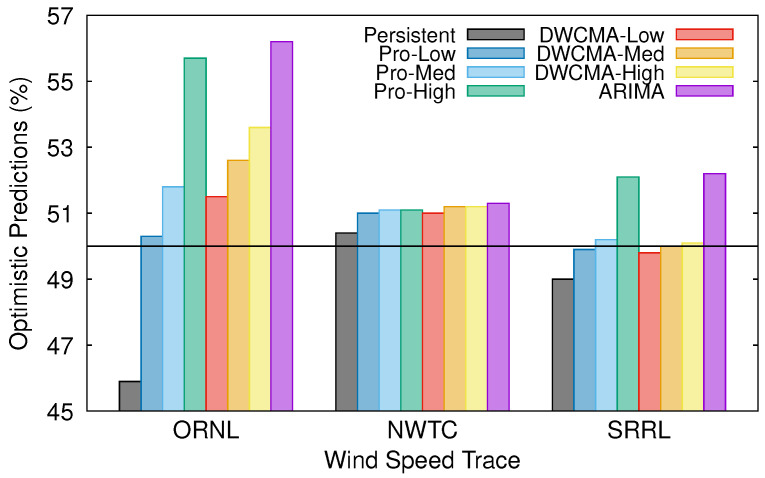
Percentage of optimistic predictions.

In this section, we check whether the selected forecasting schemes tend to overestimate (or underestimate) future wind speed. Clearly, if the actual wind speed in a given timeslot is lower than the estimated one, then the prediction was too optimistic and the energy manager could have overestimated future energy availability, thus increasing the risk of suffering an energy shortage. Conversely, if the actual wind speed is higher than the predicted one, then the prediction was excessively pessimistic and the available energy at the EH node would have been underestimated. Figure 5 shows the percentage of optimistic predictions that were obtained with each model and wind speed trace. Noticeably, all of the forecasting schemes, except the persistence one, are moderately optimistic. Additionally, note that Pro-Energy and D-WCMA schemes both tend to be more optimistic when they are configured with higher overhead settings.

We have also separately evaluated the accuracy of optimistic and pessimistic predictions. Table 2 shows the MAE for both optimistic and pessimistic predictions that were obtained with the persistent model and with those models more inclined to be optimistic (the ARIMA-based one and Pro-Energy and D-WCMA with high overload). Note that, although the persistent model tends to underestimate future wind speed, its optimistic predictions for the ORNL and SRRL traces are quite less accurate than the pessimistic ones. On the contrary, the rather optimistic Pro-Energy, D-WCMA, and ARIMA models provide optimistic predictions that are significantly more precise than the pessimistic ones. Consequently, the possibility of suffering an energy shortage is reduced when using one of these models.

### 6.3. Computational and Memory Overhead

The proposed ARIMA model is able to compute the predicted value just performing a few simple operations, as shown in Section 5.2. In particular, the forecast involves performing one subtraction to compute the last differenced value (step 2), three subtractions and two multiplications to compute the predicted differenced value for the ARIMA (0,1,2) model (step 3), or two subtractions and two multiplications for the ARIMA (1,1,1) model, and one final sum to obtain the predicted wind speed value (step 4). In addition, this model requires updating several state variables (steps 5–12), including the estimations of the first three autocovariance coefficients (steps 7–9), with each one involving one sum and one multiplication.

On the other hand, recall that the ARIMA parameters must be updated every six hours (just four times a day). Each update only requires performing two divisions for the ARIMA (0,1,2) model or two divisions and one subtraction for the ARIMA (1,1,1) one, as shown in Section 5.1.

Regarding the memory requirements, note that the ARIMA model just needs to store the last two wind speed samples ($s_{n}$, $s_{n-1}$), the last three differences ($▽s_{n}$, $▽s_{n-1}$, $▽s_{n-2}$), the last three predicted differences (${\hat{▽s}}_{n+1}$, ${\hat{▽s}}_{n}$, ${\hat{▽s}}_{n-1}$), the estimations of the first three autocovariance coefficients (${\hat{\gamma }}_{0}$, ${\hat{\gamma }}_{1}$, ${\hat{\gamma }}_{2}$), and the estimated ARIMA parameters (${\hat{\varphi }}_{1}$, ${\hat{\theta }}_{1}$, ${\hat{\theta }}_{2}$).

We have also calculated the amount of operations per forecast and memory requirements for both the Pro-Energy and D-WCMA models with the different overhead settings. Recall that, for each prediction, Pro-Energy demands computing the similarity of the current day with each of the profiles being stored in the pool using (6) and the average value that was observed at the future timeslot result of combining the most similar profiles using (7) and (8). Moreover, although not being taken into account when estimating the amount of operations, recall that Pro-Energy must also sort the profiles of the pool by their similarity with the current day, which involves some additional operations for each forecast. On the other hand, D-WCMA requires computing the weighting factor α and GAP parameter using (11) and (13), respectively, which involves computing several standard deviations (see Equation (12)). Some square roots must be performed to compute these standard deviations, but we assumed that the time complexity for computing a square root is comparable to that of a multiplication, thus preventing any eventual bias in favor of our proposal. Additionally, both of the techniques must also update their pool of profiles everyday.

Respecting their memory requirements, both of the techniques must store the pool of profiles, each one comprising the wind speed that was observed during each of the timeslots of the corresponding past day, and the wind speed that was observed during the timeslots of the current day. Table 3 resumes the amount of operations and memory usage estimated for each forecasting model. It is assumed that the float numbers are saved as 32 bit values. Definitely, the proposed ARIMA model provides accurate enough short-term forecasts with a very low memory usage and performing much less operations.

## 7. Conclusions

This paper presents a new ARIMA-based forecasting model to predict wind speed at short-term horizons (from 10 min to 1 h) especially tailored for resource-constrained devices with limited hardware capacities, such as the nodes of a WSN. In particular, we found that the wind speed time series can be alternatively well described by either an ARIMA (1,1,1) or an ARIMA (0,1,2) process, depending on the relative weight of their two first autocorrelation coefficients. Consequently, the proposed model estimates these coefficients and selects the most adequate ARIMA model and ARIMA coefficients for a given wind speed series periodically.

The performance results obtained using real data sets show that the proposed ARIMA model provides accurate forecasts in all of the considered scenarios using a very small amount of memory and just performing a few low complexity operations. Moreover, the management and setup of the proposed model is very simple since, unlike most of the forecasting models that require the careful configuration of several parameters to guarantee acceptable wind speed predictions, it is able to dynamically adapt to changing wind conditions and/or locations without requiring any particular reconfiguration for each different scenario.

## Figures and Tables

**Table 1:** Pro-Energy and Dynamic Weather Condition Moving Average (D-WCMA) settings.

	Pro-Energy			D-WCMA	
Overhead	*D*	*K*	*P*	*D*	*K*
Low	30	2	1	10	2
Medium	60	3	2	20	3
High	90	5	5	40	5

**Table 2:** MAE for optimistic and pessimistic predictions (in m/s).

		ORNL		NWTC		SRRL	
Model	Horizon	Opt-MAE	Pes-MAE	Opt-MAE	Pes-MAE	Opt-MAE	Pes-MAE
Persistent	10	0.2308	0.1907	0.4813	0.4966	0.5053	0.4876
	20	0.2722	0.2309	0.6531	0.6671	0.6926	0.6697
	30	0.2977	0.2543	0.7644	0.7791	0.8039	0.7809
	40	0.3185	0.2714	0.8496	0.8620	0.9001	0.8599
	50	0.3354	0.2883	0.9193	0.9296	0.9720	0.9341
	60	0.3515	0.3009	0.9802	0.9852	1.0358	0.9917
Pro-Energy (High)	10	0.1773	0.2320	0.4597	0.5112	0.4616	0.5231
	20	0.2059	0.2786	0.5989	0.6938	0.6148	0.7178
	30	0.2246	0.3050	0.6865	0.8068	0.7093	0.8275
	40	0.2383	0.3268	0.7483	0.8936	0.7799	0.9128
	50	0.2495	0.3447	0.7939	0.9621	0.8343	0.9859
	60	0.2616	0.3576	0.8303	1.0249	0.8839	1.0353
D-WCMA (High)	10	0.1863	0.2200	0.5068	0.5319	0.5279	0.5319
	20	0.2207	0.2545	0.6519	0.6818	0.6859	0.6824
	30	0.2433	0.2765	0.7501	0.7821	0.7876	0.7819
	40	0.2607	0.2944	0.8261	0.8603	0.8709	0.8591
	50	0.2763	0.3089	0.8932	0.9225	0.9403	0.9228
	60	0.2897	0.3228	0.9501	0.9762	0.9995	0.9748
ARIMA	10	0.1732	0.2321	0.4678	0.4991	0.4685	0.5191
	20	0.2077	0.2703	0.6258	0.6647	0.6367	0.7061
	30	0.2296	0.2956	0.7266	0.7727	0.7430	0.8174
	40	0.2485	0.3137	0.8068	0.8488	0.8309	0.9003
	50	0.2646	0.3312	0.8708	0.9134	0.9012	0.9742
	60	0.2786	0.3470	0.9262	0.9655	0.9617	1.0323

**Table 3:** Number of operations and memory overhead (in bytes).

	Op. per Day		Op. per Prediction		
Model	Add/Sub	Mul/Div	Add/Sub	Mul/Div	Memory
ARIMA (1,1,1)	4	8	7	5	56
ARIMA (0,1,2)	0	8	8	5	56
Pro-Low	Profiles Pool Update		121	32	17,856
Pro-Med			367	67	35,136
Pro-High			916	103	52,416
DWCMA-Low	Profiles Pool Update		95	37	6336
DWCMA-Med			206	60	12,096
DWCMA-High			488	106	23,616

## Data Availability

The data presented in this study are available on request from the corresponding author. The data are not publicly available due to privacy reasons.

## Abbreviations

The following abbreviations are used in this manuscript:

WSN	Wireless Sensor Network
EH	Energy Harvesting
NWP	Numerical Weather Prediction
ARIMA	AutoRegressive Integrated Moving Average
ACF	AutoCorrelation Function
PACF	Partial AutoCorrelation Function
MAE	Mean Absolute Error
